# *Trichuris trichiura* isolated from *Macaca sylvanus*: morphological, biometrical, and molecular study

**DOI:** 10.1186/s12917-020-02661-4

**Published:** 2020-11-17

**Authors:** Julia Rivero, Ángela María García-Sánchez, Antonio Zurita, Cristina Cutillas, Rocío Callejón

**Affiliations:** Department of Microbiology and Parasitology, Faculty of Pharmacy, University of Seville, Professor García González 2, 41012 Seville, Spain

**Keywords:** Barbary macaque, *Macaca sylvanus*, Mitochondrial DNA, *Trichuris trichiura*, Zoonosis

## Abstract

**Background:**

Recent studies have reported the existence of a *Trichuris* species complex parasitizing primate. Nevertheless, the genetic and evolutionary relationship between *Trichuris* spp. parasitizing humans and Non-Human Primates (NHP) is poorly understood. The hypothesised existence of different species of *Trichuris* in primates opens the possibility to evaluate these primates as reservoir hosts of human trichuriasis and other putative new species of whipworms.

**Results:**

In this paper, we carried out a morphological, biometrical and molecular study of *Trichuris* population parasitizing *Macaca sylvanus* from Spain based on traditional morpho-biometrical methods, PCA analysis and ribosomal (ITS2) and mitochondrial (*cox*1 and *co*b) DNA sequencing. Morphological results revealed that *Trichuris* sp. from *M. sylvanus* is *Trichuris trichiura*. Ribosomal datasets revealed that phylogenetic relationships of populations of *Trichuris* sp. from *M. sylvanus* were unresolved. The phylogeny inferred on mitochondrial datasets (partitioned and concatenated) revealed similar topologies; Thus, phylogenetic trees supported the existence of clear molecular differentiation between individuals of *Trichuris* sp. from *M. sylvanus* appearing in two different subclades.

**Conclusions:**

Based on morphological parameters, biometrical measurements, and molecular sequence analysis, we conclude that the whipworms isolated from *M. sylvanus* were *T. trichiura*. Further, the evolutionary relationship showed that these worms belonged to two genotypes within the *T. trichiura* lineage. Since *T. trichiura* is of public health importance, it is important to carry out further studies to improve the understanding of its hosts range, evolution and phylogeography.

## Background

*Trichuris* species are nematodes belonging to Order Trichocephalida (Class Enoplea) and they parasitize the caecum of different hosts. For many years, *Trichuris trichiura* Linnaeus, 1771 was considered as the whipworm present in humans and Non-Human-Primates (NHP). Until now, it is known that several whipworm species are able to parasitize humans: *T. trichiura* (human whipworm), *Trichuris suis* Schrank, 1788 (pig whipworm) and *Trichuris vulpis* Froelich, 1789 (dog whipworm), but only *T. trichiura* has been considered for many years to be the specific whipworm of primates.

Whipworms’ genetic and evolutionary relationship between human and NHP is poorly understood. Moreover, given the phenotypic plasticity of these parasites themselves: host-induced variation, lack of morphological characteristics, and overlap between species in morphological characteristics, it is very difficult to distinguish among closely related *Trichuris* species [1–4].

Traditionally, the research on *T. trichiura* from humans and NHP had its main objective on differentiating this species from *T. suis* found in pigs [5–9]. Morphological studies of *Trichuris* isolated from primates and humans concluded that the species infecting these hosts is the same, despite slight morphological variations observed using scanning electron microscopy [6]. However, these studies were based on a few morphological features such as the total length or spicule length, but not on discriminative analysis of many morpho-biometrical significant parameters using statistical tests. Ravasi et al. [10] carried out a study to discriminate parasite species from human and NHP using exclusively molecular techniques. Thus, these authors [10] suggested the need for morphological analysis of *Trichuris* sp. adult collected from *Papio ursinus* (Chacma baboon) from South Africa to determine whether the genetic lineages corresponded with different morphological species. It seems to be a pattern of infection with different *Trichuris* species infecting host species, thus, some authors [11] concluded that it would be necessary to apply multiple genetic markers to *Trichuris* collected from humans and NHP from sympatric areas and worldwide locations. This would clarify parasite transmission routes between these primates allowing the implementation of appropriate control and prevention measures [11].

Hawash et al. [12] suggested the existence of a *Trichuris* species complex in primates and pigs based on complete mitochondrial genome analysis. Recently, Cutillas et al. [13] proposed a new species, *Trichuris colobae,* from *Colobus guereza kikuyensis,* which is distinguished from *T. suis* from pigs and *T. trichiura* from humans. Afterward, Callejón et al. [14] reported a morpho-biometric study showing the new species *Trichuris ursinus* from another NHP (*P. ursinus*) that differed significantly from *T. trichiura* (nine different characters) and *T. colobae* (six different characters). Furthermore, *T. ursinus* showed features close to *T. suis* (only three different characters). In all these studies, *Trichuris* specimens were measured according to parameters reported by Spakulová and Lýsek [15], Suriano and Navone [16] and Robles et al. [17], who summarized the morpho-biometric parameters used in recent years.

The Barbary macaque or Gibraltar macaque (*Macaca sylvanus*) is the only member of this genus found outside Asia, distributed in Africa, North of the Sahara Desert, and the only NHP to occur in Europe (Rock of Gibraltar) [18, 19]. In early times, it was widespread throughout North Africa from Libya to Morocco, but its current distribution is limited to patches of forest and scrub in Algeria and Morocco [19–22]. A long-established introduced Barbary macaque population lives in Gibraltar [23–25].

To date, morphological and molecular studies of the *Trichuris* populations of *M. sylvanus* have not been carried out. Hence, the main objective of this work is to determine the morphologic, biometric and molecular features of *Trichuris* sp. parasitizing *M. sylvanus* from Zoo Castellar (Spain) in order to: (i) identify at species level adult parasites of these specimens and (ii) to evaluate genetic variation and evolutionary relationships between *Trichuris* spp. from NHP and humans.

## Results

### Morphological and biometrical description

#### General

The anterior part of the body is long, narrow, tapered and whip-like; the posterior part of the body broad and handle-like. The cuticle has a fine transversal striation. The bacillary band is located laterally in the anterior portion of the body.

#### Male

**Fig. 1:**
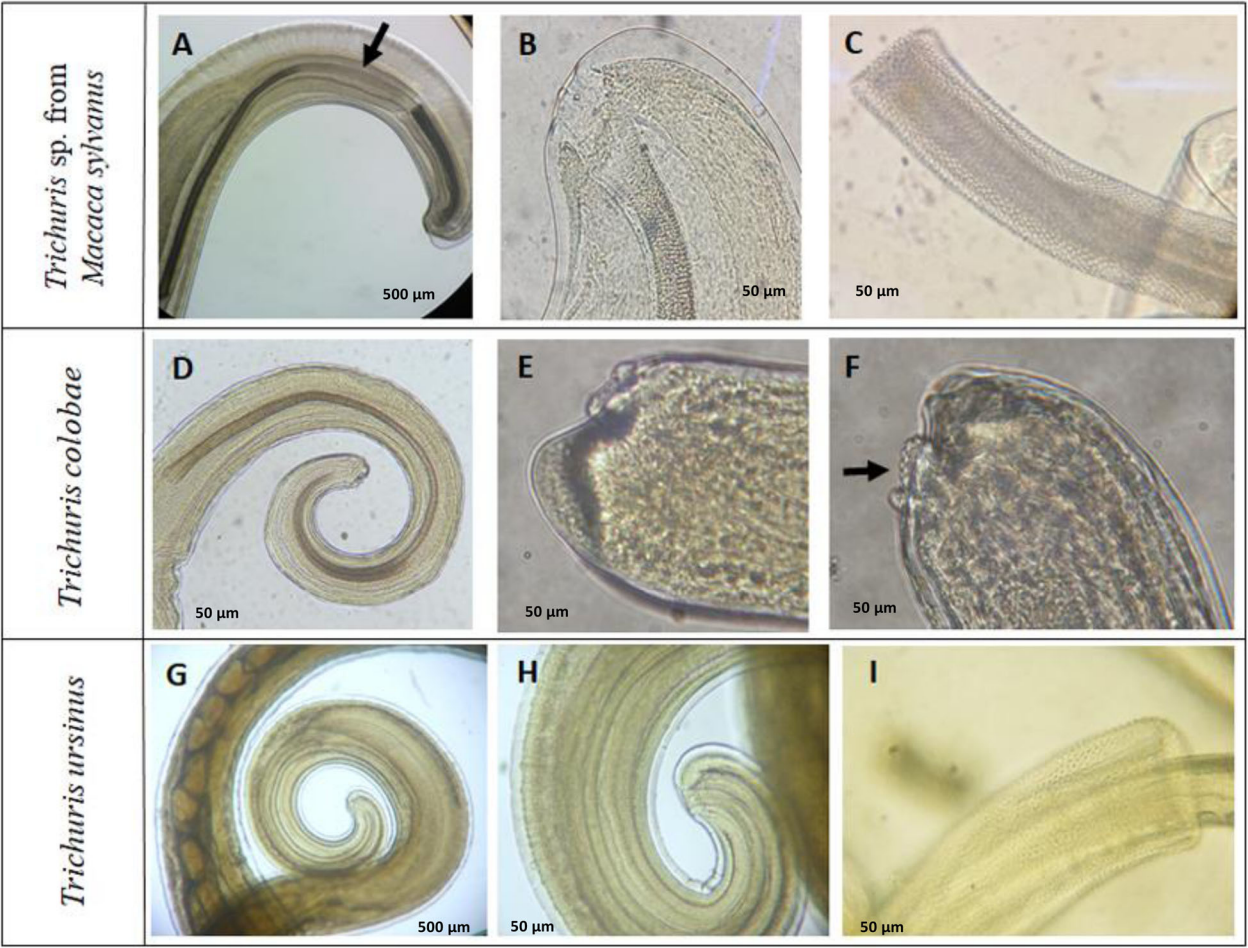
Morphology of males of *Trichuris* sp. from primates. *Trichuris* sp. from *M. sylvanus* (**a**-**c**), *Trichuris colobae* [13] (**d**-**f**) and *Trichuris ursinus* [14] (**g**-**i**). **a**-**b** Posterior end showing spicule (arrowed) and spicule sheath. **c** posterior end, spiny spicule sheath and cloaca. **d** Posterior end with spicule invaginated. **e**-**f** Posterior end showing cluster of papillae (arrowed). **g**-**h**: Posterior end with spicule invaginated. **i** spiny spicule sheath

**Fig. 2:**
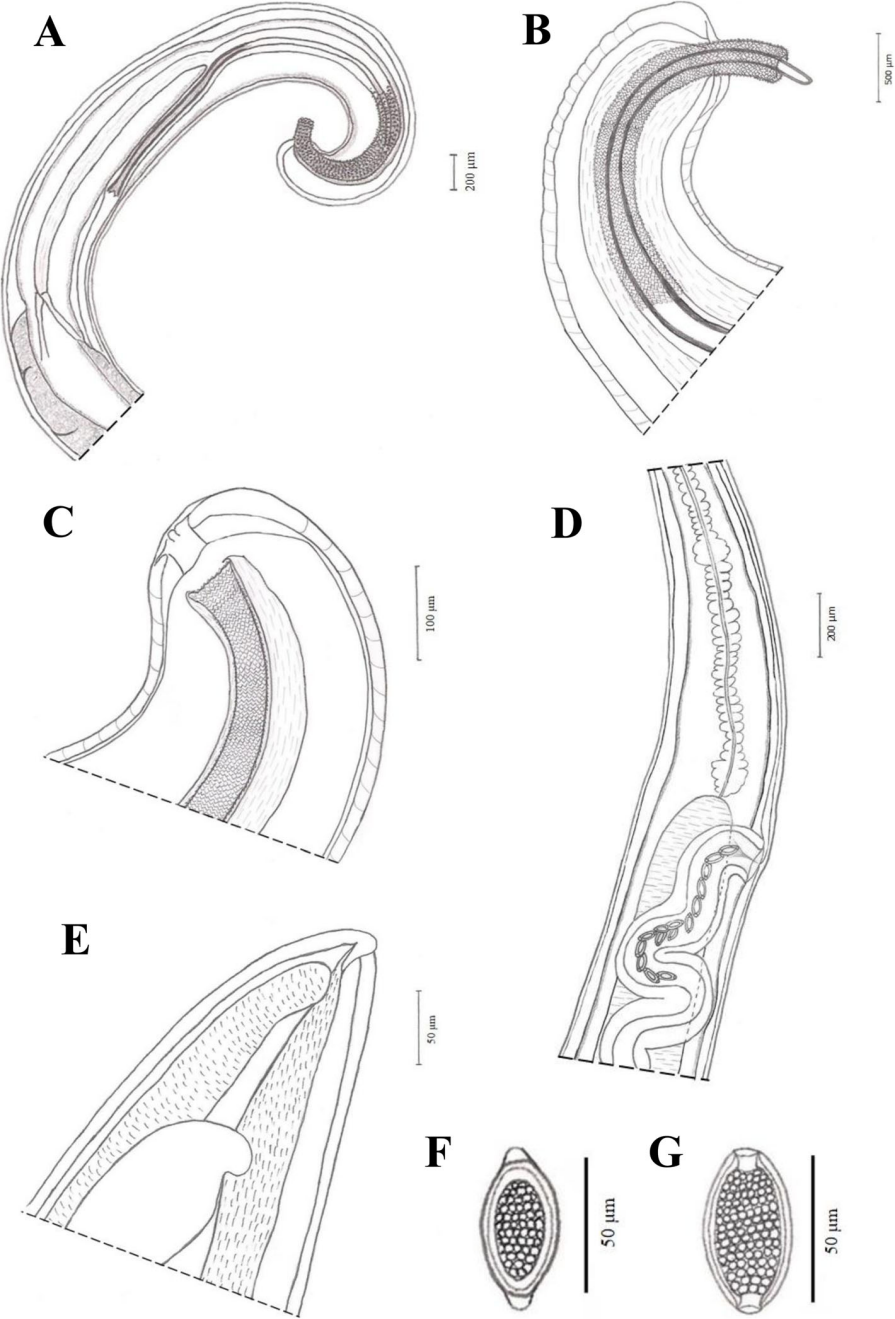
Drawings of *Trichuris* sp. from *Macaca sylvanus*. **a** Male, posterior end, spiny spicule sheath, spicule, spicule tube and proximal and distal cloacal tube, lateral view. **b** Male, detail of the posterior end, lateral view. **c** Male, cloaca subterminal with one pair of pericloacal papilla, lateral view. **d** Female, esophagus-intestine junction, vulva and vagina, lateral view. **e** Female, posterior end, lateral view. **f** and **g** Eggs

The proximal cloacal tube is wide and continued with the distal cloacal tube that contains the spicule, which projected into the anterior portion of the body in a spicule tube (Figs. 1a and 2a). The spicule was observed to have two more chitinized extreme zones and a lighter central part (Figs. 1a and 2a). Spicule sheath was cylindrical without a distal bulb (Figs. 1c and 2a-b) and with triangular spines distributed from the proximal to distal portion (Figs. 1c and 2b). The testis ends near the union of the ejaculator duct and intestine. The cloaca with anus subterminal with one pair of paracloacal papillae not ornamented (Figs. 1b and 2c) was observed when the spicule sheath was invaginated. No cluster of papillae was observed.

#### Female

**Fig. 3:**
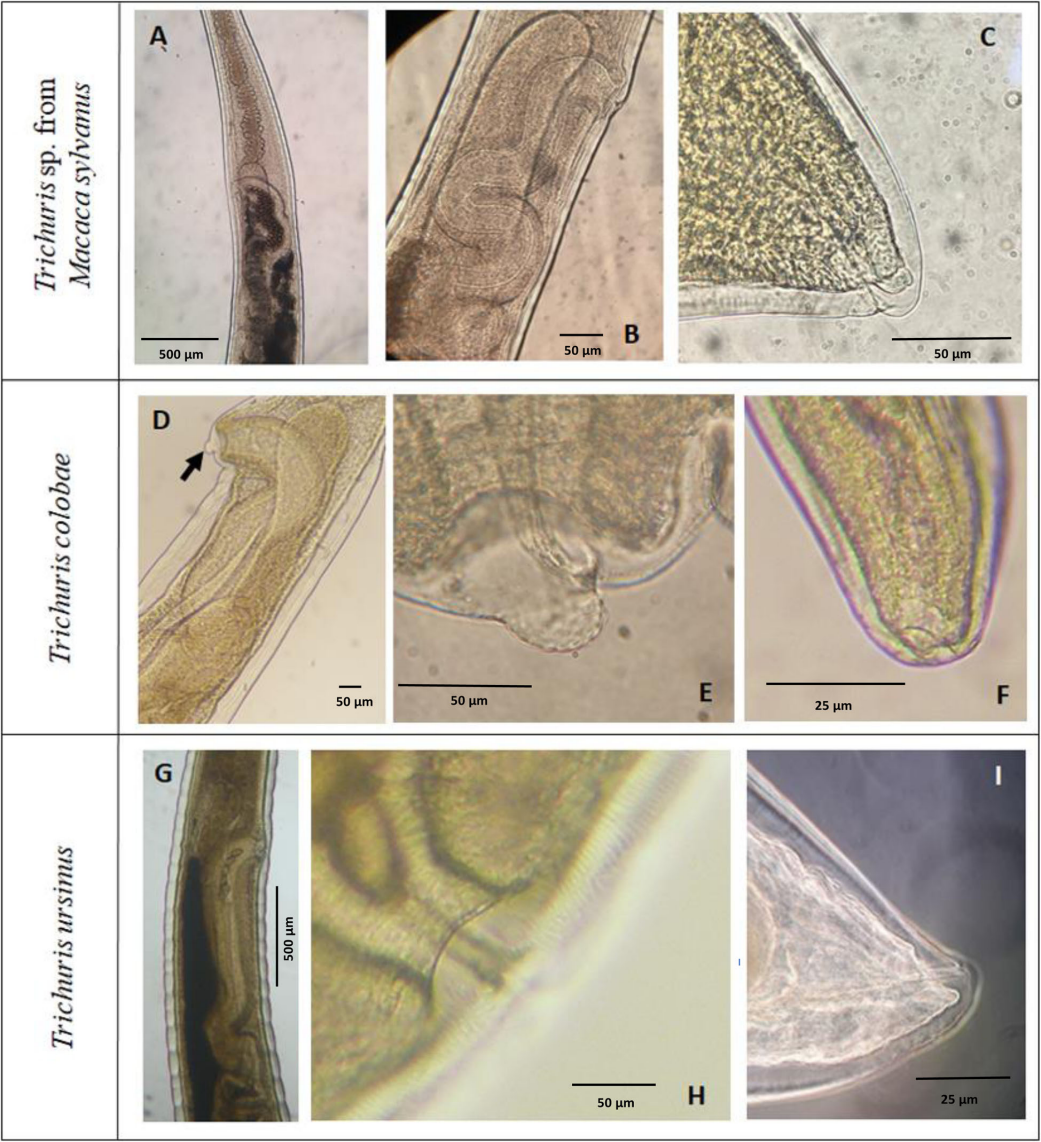
Morphology of females of *Trichuris* sp. from primates. Morphology of females of *Trichuris* sp. from primates. *Trichuris* sp. from *M. sylvanus* (**a**-**c**), *Trichuris colobae* [13] (**d**-**f**) and *Trichuris ursinus* [14] (**g**-**i**). **a** Stychocites, esophagus-intestine junction and vagina. **b** Vulva and vagina. **c** Anus subterminal. **d** Vagina and like-crater vulva (arrowed). **e** Vulva. **f** Anus. **g** Vagina and vulva. **h** Vulva. **i** Anus

A non-protrusive vulva without ornamentation was located at esophagus-intestinal junction level (Figs. 3a-b and 2d) and a short and straight vagina continuing with circumvolutions (Figs. 3a-b and2d) without papillae. The anus was subterminal (Figs. 3b and 2e). In addition, two different types of eggs were found in feces of macaque (Figs. 2f-g) which measurements ranged 60–50.6 × 38.4–23.9 μm.

Morphological results revealed that the *Trichuris* populations of *M. sylvanus* in Spain correspond to *T. trichiura*.

The comparative morphological study with other males of *Trichuris* species from primates revealed a typical spicule in *Trichuris* sp. from macaque presenting a central clear part (Fig. 1a, arrowed, 2a) that was not present in that of *T. colobae* and *T. ursinus* (Fig. 1d and g-h). In addition, the cluster of typical papillae was only present in *T. colobae* (Figs. 1e-f). Females of these species appeared to have a non-protrusive vulva (*Trichuris* sp. and *T. ursinus*) (Fig. 3b, e and h) and a vulva like a crater with papillae in *T. colobae* (Figs. 3D-E). The vagina was very long and straight in *T. ursinus* (Fig. 3g) but appeared with circumvolutions in *Trichuris* sp. from macaque (Figs. 2d, 3a-b).

The preliminary biometrical study carried out in males and females of *Trichuris* sp. from *M. sylvanus* revealed data of 15 different parameters (see Additional files 1 and 2).

The Student’s t test was carried out considering the two different populations according to the two observed genetic lineages: TT2a and TT2c. *P* values for all measurements were higher than 0.05, thus, no any significant differences between both populations were detected (Tables 1 and 2).

**Table 1:** Biometrical and morphological data of 33 males of *Trichuris* sp. isolated from *M. sylvanus*. TM = *Trichuris* male. EL/BL = Esophagus length/Body length. Б: standard deviation. X: arithmetic mean. Min: Minimum value. Max: Maximum value. All measurements are in millimetres

Lineage TT2a	Total length	Esophagus length	Body length	Ratio EL/BL	Wide body	Spicule length	Spicular length sheath	Spicular tube	Proximal cloacal tube	Pericloacal papillae	Spicular sheath with spines	Distal bulb	Cluster with papillae
TM3	38	24	14	1.71	0.64	2.46	0.86	Present	Present	Not visible	Present	Not present	Not present
TM5	39	24	15	1.60	0.75		0.84	Present	Present	Not visible	Present	Not present	Not present
TM8	41	28	13	2.15	0.74	2.87	0.91	Present	Present	Not visible	Present	Not present	Not present
TM18	31	19	12	1.58	0.63	2.77	0.97	Present	Present	Present	Present	Not present	Not present
MIN	31	19	12	1.58	0.63	2.46	0.84						
MAX	41	28	15	2.15	0.75	2.87	0.97						
X	37.25	23.75	13.50	1.76	0.69	2.70	0.90						
Б	4.35	3.69	1.29	0.27	0.06	0.21	0.06						
Lineage TT2c													
TM1	35	22	13	1.69	0.69	2.75	0.87	Present	Present	Not visible	Present	Not present	Not present
TM2	34	23	11	2.09	0.67	2.56	0.90	Present	Present	Not visible	Present	Not present	Not present
TM4	36	22	14	1.57	0.63	2.82	0.81	Present	Present	Not visible	Present	Not present	Not present
TM6	32	21	11	1.91	0.66	2.46	0.73	Present	Present	Not visible	Present	Not present	Not present
TM7	31	20	11	1.82	0.50	2.33	1.12	Present	Present	Present	Present	Not present	Not present
TM9	34	22	12	1.83	0.54	2.55	1.01	Present	Present	Present	Present	Not present	Not present
TM10	35	21	14	1.50	0.63	2.61	0.70	Present	Present	Not visible	Present	Not present	Not present
TM11	35	22	13	1.69	0.59	2.51	0.47	Present	Present	Present	Present	Not present	Not present
TM12	35.50	24.50	11	2.23	0.67	2.48	1.02	Present	Present	Present	Present	Not present	Not present
TM13	37	24	13	1.85	0.7	2.73	0.96	Present	Present	Not visible	Present	Not present	Not present
TM14	39	29	10	2.90	0.61	2.66	0.85	Present	Present	Not visible	Present	Not present	Not present
TM15	27	16	11	1.45	0.47	2.16	0.91	Present	Present	Not visible	Present	Not present	Not present
TM16	36	23	13	1.77	0.57	2.71	1.16	Present	Present	Not visible	Present	Not present	Not present
TM17	32	21	11	1.91	0.65	2.53	0.77	Present	Present	Not visible	Present	Not present	Not present
TM19	35	23	12	1.92	0.62	2.64	0.86	Present	Present	Not visible	Present	Not present	Not present
TM20	33	21	12	1.75	0.65	2.63	0.95	Present	Present	Not visible	Present	Not present	Not present
TM21	32	21	11	1.91	0.63	2.88	0.75	Present	Present	Not visible	Present	Not present	Not present
TM22	35	21	14	1.50	0.65	2.68	0.64	Present	Present	Not visible	Present	Not present	Not present
TM23	33	21	12	1.75	0.67	2	0.63	Present	Present	Not visible	Present	Not present	Not present
TM24	31	20	11	1.82	0.69	2.55	0.79	Present	Present	Not visible	Present	Not present	Not present
TM25	32	20.5	11.50	1.78	0.67	2.77	0.88	Present	Present	Not visible	Present	Not present	Not present
TM26	34	23	11	2.09	0.66	2.39	1.10	Present	Present	Present	Present	Not present	Not present
TM27	30	20	10	2.00	0.5	2.19	1.01	Present	Present	Present	Present	Not present	Not present
TM28			11.50		0.57	2.81	0.88	Present	Present	Not visible	Present	Not present	Not present
TM29	30	19	11	1.73	0.53	2.22	0.73	Present	Present	Not visible	Present	Not present	Not present
TM30	34	23	11	2.09	0.62	2.53	0.57	Present	Present	Not visible	Present	Not present	Not present
TM31	31	20	11	1.82	0.56	2.39	0.90	Present	Present	Not visible	Present	Not present	Not present
TM32	34	22	12	1.83	0.7	2.71	0.94	Present	Present	Present	Present	Not present	Not present
TM33	35	23	12	1.92	0.59	2.46	0.87	Present	Present	Not visible	Present	Not present	Not present
MIN	27	16	10	1.45	0.47	2	0.47						
MAX	39	29	14	2.90	0.70	2.88	1.16						
X	33.48	21.71	11.70	1.88	0.61	2.54	0.85						
Б	2.50	2.22	1.12	0.28	0.06	0.21	0.16

**Table 2:** Biometrical and morphological data of 32 females of *Trichuris* sp. isolated from *M. sylvanus*. TF = *Trichuris* female. Б: standard deviation. X: arithmetic mean. Min: Minimum value. Max: Maximum value. All measurements are in millimetres

Lineage TT2a	Total length	Esophagus length	Body length	Ratio EL/BL	Wide body	Vulvar diameter	Vulva non protusive	Vulva with papillae or ornamentation	Vagina with circumvolutions	Anus subterminal
TF10	27	17	10	1.70	0.6	0.07	Present	No	Present	Present
TF27	35	22	13	1.69	0.66	0.09	Present	No	Present	Present
TF30	36	24	12	2.00	0.69	0.07	Present	No	Present	Present
TF32	32	22	10	2.20	0.61	0.08	Present	No	Present	Present
MIN	27	17	10	1.69	0.60	0.07				
MAX	36	24	13	2.20	0.69	0.09				
X	32.50	21	11.33	1.91	0.64	0.08				
Б	4.04	2.99	1.50	0.25	0.04	0.01				
Lineage TT2c										
TF1	33	23	10	2.30	0.69	0.06	Present	No	Present	Present
TF2	35	23	12	1.92	0.67	0.05	Present	No	Present	Present
TF3	33	21	11	1.91	0.7	0.06	Present	No	Present	Present
TF4	35	22	13	1.69	0.75	0.05	Present	No	Present	Present
TF5	32	21	11	1.91	0.7	0.06	Present	No	Present	Present
TF6	32.50	21	11.50	1.83	0.76	0.06	Present	No	Present	Present
TF7	37	25.50	11.50	2.22	0.72	0.05	Present	No	Present	Present
TF8	36	22	14	1.57	0.52	0.05	Present	No	Present	Present
TF9	33	22	11	2.00	0.69	0.05	Present	No	Present	Present
TF11	37	26	11	2.36	0.74	0.05	Present	No	Present	Present
TF12	32	21	11	1.91	0.66	0.06	Present	No	Present	Present
TF13	32	22	10	2.20	0.57	0.05	Present	No	Present	Present
TF14	37	24	13	1.85	0.71	0.06	Present	No	Present	Present
TF15	38	25	13	1.92	0.65	0.06	Present	No	Present	Present
TF16	31	21	10	2.10	0.63	0.05	Present	No	Present	Present
TF17	34	22	12	1.83	0.71	0.05	Present	No	Present	Present
TF18	34	22	12	1.83	0.71	0.05	Present	No	Present	Present
TF19	33	22	11	2.00	0.73		Present	No	Present	Present
TF20	34	22	12	1.83	0.59	0.06	Present	No	Present	Present
TF21	33	23	10	2.30	0.83	0.05	Present	No	Present	Present
TF22	34	24	10	2.40	0.69	0.06	Present	No	Present	Present
TF23	30	20	10	2.00	0.62	0.07	Present	No	Present	Present
TF24	36	23	13	1.77	0.69	0.05	Present	No	Present	Present
TF25	32	21	11	1.91	0.77	0.05	Present	No	Present	Present
TF26	37	26	11	2.36	0.71	0.06	Present	No	Present	Present
TF28	34	23	11	2.09	0.7	0.06	Present	No	Present	Present
TF29	37	26	11	2.36	0.79	0.06	Present	No	Present	Present
TF31	35	24	11	2.18	0.61	0.07	Present	No	Present	Present
MIN	30	20	10	1.57	0.52	0.05				
MAX	38	26	14	2.40	0.83	0.07				
X	34.15	22.78	11.40	2.02	0.69	0.06				
Б	2.10	1.72	1.09	0.23	0.07	0.01				

**Fig. 4:**
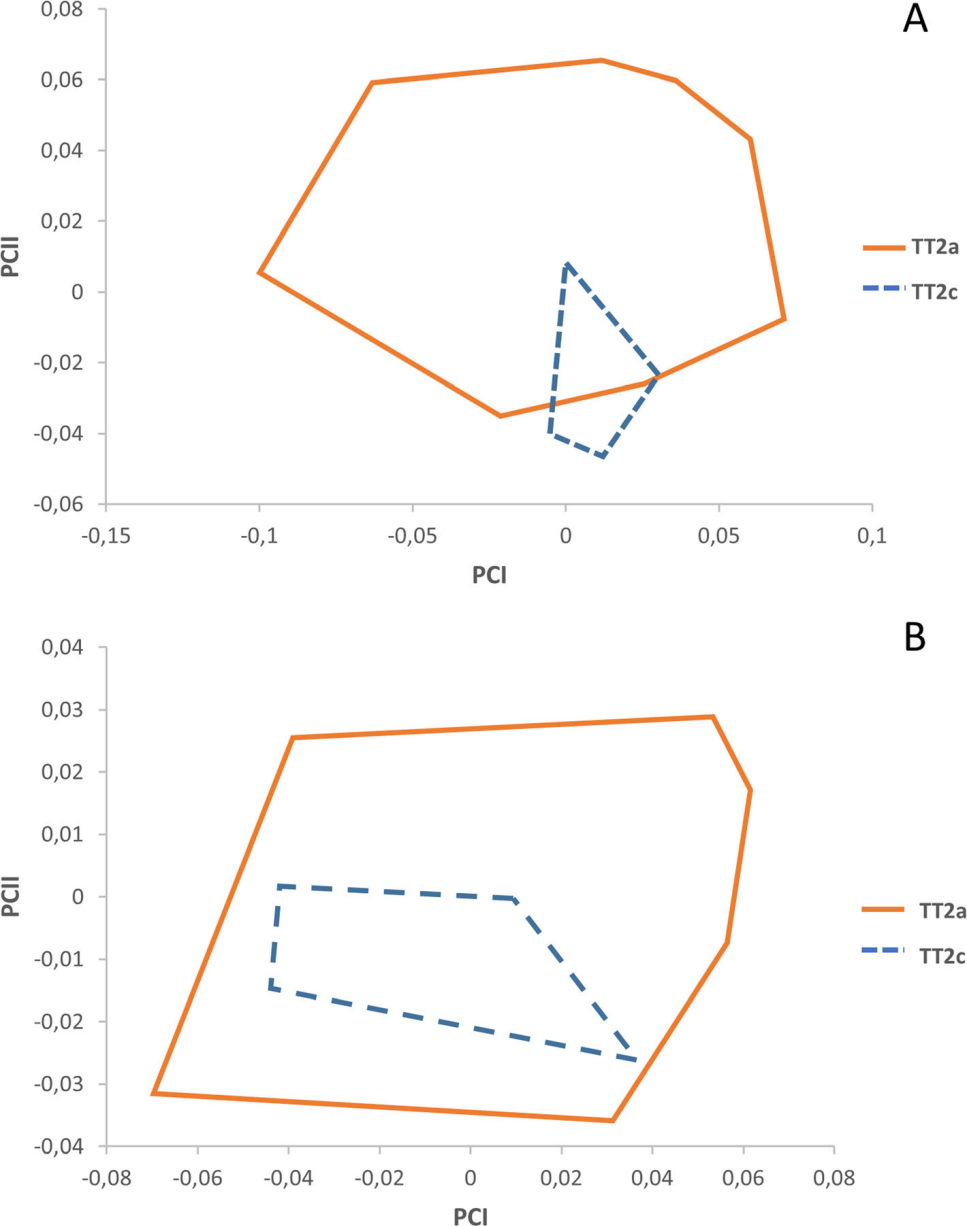
Factor maps corresponding to adult *Trichuris* sp. Samples are projected onto the first (PC1) and second (PC2) principal components. Each group is represented by its perimeter. **a** Males factor map. PC1: 57% and PC2: 35%. **b** Females factor map. PC1: 81% and PC2: 18%

The resulting factor maps for male and female populations of *Trichuris* sp. adults are represented in Fig. 4a and b, respectively. Both male populations slightly overlapped while the female communities appeared 100% overlapped. Males factor map was PC1: 57% and PC2: 35%, while females factor map was PC1: 81% and PC2: 18%.

### Annotation and features of ribosomal and mitochondrial genomes

The ITS2 region was amplified from the genomic DNA of 13 *Trichuris* sp. specimens from *M. sylvanus* from Spain revealing 7 haplotypes. These sequences were 587 base pairs (bp) in length and their G + C content ranged from 66.1–66.6%. *Cox*1 mitochondrial DNA (mtDNA) partial gene was sequenced from 43 individuals revealing the presence of 4 haplotypes. *Cox*1 sequences were 370 bp in length and their G + C content ranged from 38 to 39.2%. The *co*b partial gene was amplified from 13 specimens of *Trichuris* sp. revealing the presence of 4 haplotypes. The sequences (520 bp) revealed a G + C content ranging from 30.2 to 31.0%.

The datasets generated and analyzed during the current study are available in the GenBank™, EMBL and DDBJ repository, [Accession numbers: LR130781–4, LR132031–4, LR535742, LR535746–51] (Table 3).

**Table 3:** Sequences of *Trichuris* spp. and outgroups species obtained from GenBank and used for phylogenetic analysis

Species	Host species/Family	Geographical origin	Marker	Accession number
*Trichuris colobae*	*Colobus guereza kikuyensis*/ Cercopithecidae	Spain (Europe)	***Cox*1**	HE653116 HE653118
*Trichuris* sp.	*Colobus guereza kikuyensis*/Cercopithecidae	Czech Republic (Europe)		JF690968
	*Homo sapiens*/Hominidae	Czech Republic (Europe)		JF690962
	*Macaca fuscata*/ Cercopithecidae	Italy (Europe)		MK762905 MK762906 MK762908 MK762909 MK762915 MK762919 MK762920 MK762921
	***Macaca sylvanus/ Cercopithecidae***	**Spain** (Europe)		**LR130781**^**a**^ **LR130782**^**a**^ **LR130783**^**a**^ **LR130784**^**a**^
	*Papio anubis*/ Cercopithecidae	USA (North America)		KT449825
	*Papio hamadryas*/ Cercopithecidae	Czech Republic (Europe)		JF690963
	*Papio hamadryas*/ Cercopithecidae	Denmark (Europe)		KT449824
*Trichuris trichiura*	*Homo sapiens*/Hominidae	China (Asia)		GU385218
		Japan (Asia)		AP017704
		Uganda (Africa)		KT449826
*Trichuris suis*	*Sus scrofa domestica*/Suidae	Spain (Europe)		HE653124
		Denmark (Europe)		KT449822
		Uganda (Africa)		KT449823
		China (Asia)		GU070737 HQ204208 HQ204209 HQ204210
	*Sus scrofa scrofa*/Suidae	Spain (Europe)		HE653127
*Trichuris ursinus*	*Papio ursinus*/ Cercopithecidae	South Africa (Africa)		LT627353
*Trichinella spiralis*		USA (North America)		AF293969
*Trichinella pseudospiralis*		Australia (Oceania)		KM357411
*Trichuris colobae*	*Colobus guereza kikuyensis*/ Cercopithecidae	Spain (Europe)	***Co*b**	LM994704
*Trichuris* sp.	*Macaca fuscata*/ Cercopithecidae	Italy (Europe)		MK914550 MK914551 MK914554 MK914555 MK914556 MK914557 MK914560
	***Macaca sylvanus/ Cercopithecidae***	**Spain** (Europe)		**LR132031**^**a**^ **LR132032**^**a**^ **LR132033**^**a**^ **LR132034**^**a**^
	*Papio anubis*/ Cercopithecidae	USA (North America)		KT449825
	*Papio hamadryas*/ Cercopithecidae	Denmark (Europe)		KT449824
	*Papio* sp. /Cercopithecidae	Spain (Europe)		LM994703
*Trichuris trichiura*	*Homo sapiens*/Hominidae	China (Asia)		GU385218
		Uganda (Africa)		KT449826
*Trichuris suis*	*Sus scrofa domestica*/Suidae	China (Asia)		GU070737
		Denmark (Europe)		KT449822
		Uganda (Africa)		KT449823
	*Sus scrofa scrofa*/Suidae	Spain (Europe)		LM994696
*Trichuris ursinus*	*Papio ursinus*/ Cercopithecidae	South Africa (Africa)		LT627357 LT627358 LT627359 LT627360
*Trichinella spiralis*		USA (North America)		NC_002681
*Trichinella pseudospiralis*		Australia (Oceania)		KM357411
*Trichuris colobae*	*Colobus guereza kikuyensis*/ Cercopithecidae	Spain (Europe)	**ITS2**	FM991956
*Trichuris trichiura*	*Macaca fuscata*/ Cercopithecidae	China (Asia) Japan (Asia)		AM992987
				AB586133
	*Homo sapiens*	Cameroon (Africa)		GQ301555
*Trichuris* sp.	*Chlorocebus aethiops*/ Cercopithecidae	Tanzania (Africa)		JF690949 JF690950
	*Homo sapiens*/Hominidae	Czech Republic (Europe)		JF690940
	*Macaca leonina*/ Cercopithecidae	China (Asia)		MH390365
				KT344828
	*Macaca mulatta*/ Cercopithecidae	China (Asia)		MH390367
				MH390369
	*Macaca silenus*/ Cercopithecidae	Czech Republic (Europe)		JF690945
	***Macaca sylvanus/ Cercopithecidae***	**Spain** (Europe)		**LR535742**^**a**^ **LR535746**^**a**^ **LR535747**^**a**^ **LR535748**^**a**^ **LR535749**^**a**^ **LR535750**^**a**^ **LR535751**^**a**^
	*Papio anubis*/ Cercopithecidae	Czech Republic (Europe)		JF690942
	*Papio hamadryas*/ Cercopithecidae	Czech Republic (Europe)		JF690941
	*Papio hamadryas ursinus*/ Cercopithecidae	South Africa (Africa)		GQ301551 GQ301552 GQ301553
	*Pan troglodytes*/Hominidae	Netherlands (Europe)		JF690948
*Trichuris suis*	*Sus scrofa*	Slovakia (Europe)		JF690951
		Tanzania (Africa)		JN181811
	*Sus scrofa domestica*	Spain (Europe)		AJ249966
*Trichuris ursinus*	*Papio hamadryas ursinus*/ Cercopithecidae	South Africa (Africa)		GQ301554

^a^Access number of this study

### Phylogenetic analysis

**Fig. 5:**
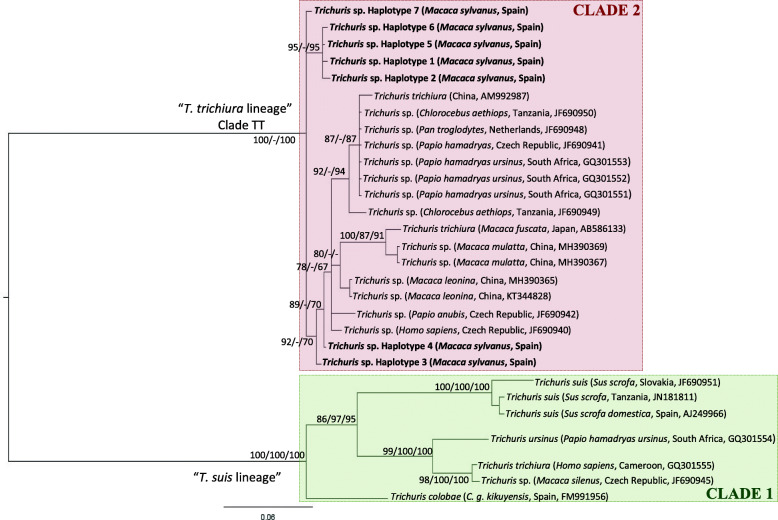
Phylogenetic tree of *Trichuris* species based on ITS2 (rDNA) inferred using Bayesian Inference. Bayesian Posterior Probabilities of clades are listed first, followed by Maximum Parsimony and Maximum Likelihood bootstrap values, respectively, for clade frequencies exceeding 65%

All trees (*cox*1, *co*b and ITS2) obtained by ML, MP and BI for *Trichuris* sp. revealed two highly supported phylogenetic clades (observed in a previous study) [26] that we named: “*T. trichiura* lineage” and “*T. suis* lineage” (Fig. 5 and Table 4). Clade 1 (“*T. suis* lineage”) included *T. colobae, T. ursinus* and *T. suis* and Clade 2 (“*T. trichiura* lineage”) included *T. trichiura* and *Trichuris* sp. from NHP corresponding to the genus *Macaca*, *Papio* and *Chlorocebus*.

**Table 4:** Monophyly based on different markers (ITS2, *cox*1, and *co*b) of selected group based on different combinations of datasets and inference methods

	Nuclear region ITS2	*Cox*1 mt gene	*Co*b mt gene	Mitochondrial genes (*cox*1 + *co*b)	Mitochondrial and nuclear markers (*cox*1, *co*b and ITS2)
Clade 1	100/100/100	76/−/93	93/69/99	92/61/100	100/−/100
Clade 2	100/−/100	73/98/80	92/−/100	90/−/100	100/100/100
Subclade TT2a	–	88/99/84	92/100/100	96/100/100	89/100/100
Subclade TT2b	–	98/100/93	92/99/99	98/100/96	–
Subclade TT2c	–	98/100/92	68/100/89	96/−/100	100/100/100
Subclade TT2d	–	−/100/−	92/100/99	100/100/100	–
Subclade TT2a + TT2b	–	–	100/96/99	70/96/84	–
Subclade TT2a + TT2b + TT2c	–	–	69/100/68	–	–
Subclade TT2c + TT2d	–	88/96/86	–	74/−/82	–
*Trichuris colobae*	–	88/100/100	–	–	–
*Trichuris ursinus*	–	–	100/100/100	–	–
*Trichuris suis*	100/100/100	−/−/100	95/99/100	98/100/100	–
*Trichuris suis* subclade 1a	–	100/100/96	91/−/63	88/100/100	–
*Trichuris suis* Subclade 1b	–	87/98/82	–	−/99/70	–
*Trichuris colobae* + *Trichuris ursinus*	–	–	−/64/−	76/−/86	−/74/62

*ML* Maximum Likelihood bootstrap, *MP* Maximum Parsimony bootstrap, *BPP* Bayesian Posterior Probability. Clade 2: *Trichuris trichiura* lineage; Clade 1: *Trichuris suis* lineage; subclade TT2a: *Trichuris* sp. from *M. sylvanus* (haplotypes 2, 3 and 4), *Trichuris* sp. from *H. sapiens* from Czech Republic; subclade TT2b: *Trichuris* sp. from *H. sapiens* and *P. anubis* from Asia and USA; subclade TT2c: *Trichuris* sp. from *M. sylvanus* (haplotypes 1 and 2), *H. sapiens* from Uganda, *P. hamadryas* from Denmark and Czech Republic, subclade TT2d*: Trichuris* sp. from *M. fuscata* from Europe

The alignment of 29 ITS2 ribosomal DNA (rDNA) sequences of *Trichuris* species from human, swine and NHP from different geographic origins yielded a dataset of 584 characters. The phylogenetic tree inferred on DatasetITS2 (Fig. 5) placed *Trichuris* spp*.* from *M. sylvanus* within “*T. trichiura* lineage” (Clade 2) without any pattern of distribution according to the host species or geographical origin. Nevertheless, *Trichuris* sp. populations from genus *Macaca* from Asia (*M. leonina*, *Macaca fuscata* and *M. mulatta*) clustered together and separated from *Trichuris* sp*.* from *M. sylvanus* from Europe (Spain) (Fig. 5). In addition, populations of *Trichuris* sp. from *M. sylvanus* appeared in different groups, out of which the haplotypes H1, H2, H5 and H6 clustered together (95% ML BV, 95% BPP), H3 and H4 clustered to the rest of the populations of *Trichuris* spp. from humans and NHP and separated from H7 (Fig. 5). Exceptionally, one individual of *T. trichiura* from *Homo sapiens* from Cameroon and one individual of *Trichuris* sp*.* from *M. silenus* from the Czech Republic were included within “*T. suis* lineage” (Clade 1)*.* The DatasetITS2 provided moderate phylogenetic resolution among most of *Trichuris* taxa regardless of inference method (Fig. 5).

**Fig. 6:**
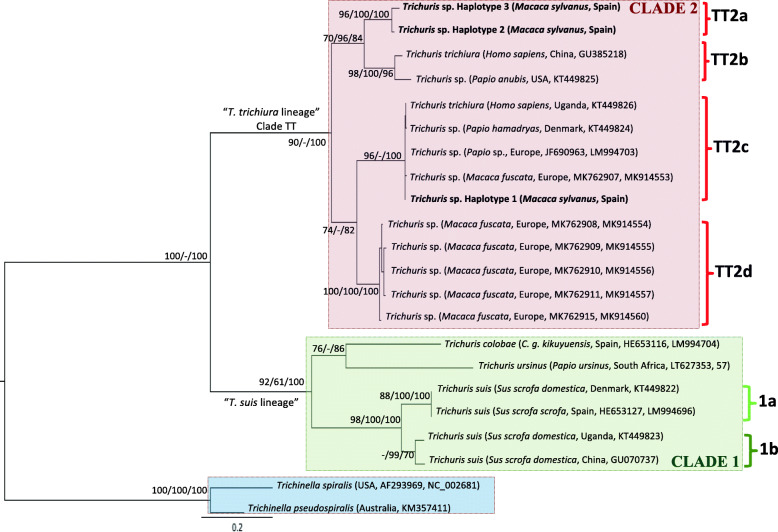
Phylogenetic tree of *Trichuris* species based on combined analysis of mitochondrial DNA (*cox*1 and *co*b) inferred using Bayesian Inference. Bayesian Posterior Probabilities of clades are listed first, followed by Maximum Parsimony and Maximum Likelihood bootstrap values, respectively, for clade frequencies exceeding 60%

The phylogeny inferred on mitochondrial datasets (partitioned and concatenated) revealed similar topologies; therefore, we assumed the concatenated tree based on mitochondrial datasets (*cox*1 and *co*b) to be the most representative (Fig. 6; Additional files 3 and 4). The concatenated dataset included 746 aligned positions and 22 taxa, including outgroups. ML, MP and BI methods showed congruence between each other revealing two main clades (corresponding with “*T. suis* lineage” and “*T. trichiura* lineage”) and respect to the sister-group relationships between *Trichuris* spp. from NHP, humans and pigs (Fig. 6 and Table 4). Four different highly supported subclades were observed within “*T. trichiura* lineage” (Clade 2): subclade TT2a including: *Trichuris* sp. from *M. sylvanus* from Spain (haplotypes 2 and 3); subclade TT2b: *T. trichiura* from *H. sapiens* from China and *Trichuris* sp. from *Papio anubis* from the USA; subclade TT2c: *Trichuris* sp. from *M. sylvanus* from Spain (majority haplotype 1), *T. trichiura* from *H. sapiens* from Uganda, *Trichuris* sp. from *Papio hamadryas* from Europe and two haplotypes of *Trichuris* sp. from *M. fuscata* from Europe; subclade TT2d: five haplotypes of *Trichuris* sp. from *M. fuscata* from Europe (Fig. 6). The phylogenetic topology revealed a sister relationship between subclades TT2a and TT2b and between subclades TT2c and TT2d with high bootstrap and BPP values (Fig. 6 and Table 4).

The multiple alignments of 33 *cox*1 nucleotide sequences (including outgroups) yielded a dataset of 301 characters. Population from *M. sylvanus* was represented by 4 haplotypes, out of which, haplotypes 3 and 4 clustered together with *Trichuris* sp. from *H. sapiens* from the Czech Republic (subclade TT2a) and separated from the main haplotype 1 and haplotype 2 (subclade TT2c) (Additional file 3 and Table 4).

The multiple alignments of 27 *co*b nucleotide sequences (including outgroups) yielded a dataset of 520 characters. Within Clade 2, population from *M. sylvanus* was represented by 4 haplotypes, out of which, haplotypes 2, 3 and 4 clustered together within subclades TT2a whereas haplotype 1 appeared within subclade TT2c (see Additional file 4).

The concatenated mitochondrial and ribosomal sequences included 1463 aligned positions and 8 taxa (outgroups not included). Phylogenetic trees again supported the existence of the two main evolutionary lineages previously recognized and the existence of clear differentiation between individuals of *Trichuris* sp. from *M. sylvanus* separated in two different subclades (TT2a and TT2c) (see Additional file 5 and Table 4).

### Comparative sequence analysis

The range of intra-population similarity of *Trichuris* sp. from *M. sylvanus* based on ITS2 rDNA sequences (seven haplotypes) was 99.6–100%. The similarity obtained within “*T. trichiura* lineage” ranged from 94.4 to 100% while within “*T. suis* lineage” this value ranged from 85.1 to 100%. When we compared ITS2 sequences corresponding with both lineages, the similarity observed ranged from 74 to 78.6%. Within “*T. trichiura* lineage”, the minimum similarity observed between *Trichuris* populations from different species of genus *Macaca* corresponded to *Trichuris* sp*.* from *M. sylvanus* from Spain and *M. fuscata* from Japan*,* and the maximum value of similarity was obtained when we compared *Trichuris* sp*.* from *M. sylvanus* and *M. leonina* from China (95.9 -99.4% respectively) (Fig. 5).

The *cox*1 sequences (four haplotypes) of *Trichuris* sp. obtained from *M. sylvanus* from Spain showed an intra-specific similarity of 83.6–100%. Thus, haplotype 1 was the main haplotype with 37 individuals showing the same *cox*1. In addition, haplotypes H1 and H2 showed a similarity percentage from 99.3 to 100% with respect to *Trichuris* sp. of *M. fuscata* from Europe and *Trichuris* sp. of *P. hamadryas* from the Czech Republic, respectively. The similarity observed within these subclades ranged from 95.6% (TT2b) to 100% (TT2a and TT2c). On the other hand, the similarity observed between these subclades ranged from 79.1% (when we compared TT2b with TT2c) to 87.0% (when we compared TT2c with TT2d). Furthermore, the similarity observed when we compared populations of *Trichuris* spp. from human and NHP with *T. suis*, *T. colobae* and *T. ursinus,* ranged from 73.4 to 80.6% (Table 5).

**Table 5:** Intra-specific and inter-specific similarity observed in *cox*1 partial sequences in *Trichuris* species isolated from different hosts

*Cox*1								
	*Trichuris* spp. (Subclade TT2a)	*Trichuris* spp. (Subclade TT2b)	*Trichuris spp. * (Subclade TT2c)	*Trichuris* spp. (Subclade TT2d) Subclade. *M. fuscata*	*T. suis* (Subclade 1b*)*	*T. suis* (Subclade 1a)	*T. colobae*	*T. ursinus*
*Trichuris* spp. (Subclade TT2a)	98–100							
*Trichuris* spp. (Subclade TT2b)	80.1–82.3	95.6–98.6						
*Trichuris* spp. (Subclade TT2c)	83.4–84.1	79.1–82.1	99.3–100					
*Trichuris* spp. (Subclade TT2d) Subclade. *M. fuscata*	84.1–86.4	81.7–83.4	85.4–87.0	97.7–99				
*T. suis* Spain (Subclade 1a)	78.4–79.2	78.4–80.6	78.1–78.7	77.4–79.7	99.7–100			
*T. suis* China (Subclade 1b)	77–78.1	76.7–79.5	77.1–78.7	75.4–78.4	90.9–92	95.3–99.5		
*T. colobae*	75.6–76.1	76.2–78.9	76.4–77.4	76.7–78.7	78.4–78.9	77.8–79.2	99.7–100	
*T. ursinus*	74.8–75.6	73.4–74.2	75.1–76.1	74.8–75.4	81.2–81.4	81.4–83.9	80.4–80.9	100

Subclade TT2a: *Macaca sylvanus* from Spain, *Homo sapiens* from Czech Republic,. Subclade TT2b: *H. sapiens* from China and Japan, *P. anubis* from USA. Subclade TT2c: *M. sylvanus* from Spain, *Macaca fuscata* from Italy, *H. sapiens* from Uganda and *P. hamadryas* from Denmark and Czech Republic. Subclade TT2d: *M. fuscata* from Italy. Subclade 1a: *Sus scrofa domestica* from Denmark and Spain and *S. s. scrofa* from Spain. Subclade 1b: *S. s. domestica* from China

The 13 *co*b sequences of *Trichuris* sp. revealed the existence of four different haplotypes corresponding to two different lineages. The intra-specific similarity between those haplotypes ranged from 84.2 to 100%, corresponding to the lowest values observed when haplotype 1 was compared with haplotypes 2, 3 and 4. The *co*b sequences similarity observed within and between subclades revealed similar results that those obtained by *cox*1 sequences (Table 6).

**Table 6:** Intra-specific and inter-specific similarity observed in *co*b partial sequences in *Trichuris* species isolated from different host

*co*b								
	*Trichuris* spp*.* (Subclade TT2a)	*Trichuris* spp. (Subclade TT2b)	*Trichuris* spp. (Subclade TT2c)	*Trichuris* spp. (subclade TT2d) *Macaca fuscata* from Europe	*T. suis* (Subclade 1b)	*T. suis* (Subclade 1a)	*T. colobae*	*T. ursinus*
*Trichuris* spp. (Subclade TT2a)	97.1–100							
*Trichuris* spp*.* (Subclade TT2b)	81.7–88.1	93.9						
*Trichuris* spp. (Subclade TT2c)	83.8–84.4	84–84.7	99.1–99.8					
*Trichuris* spp. (subclade TT2d) *Macaca fuscata* from Europe	84.7–86.7	86–87.6	86–88.2	98.7–100				
*T. suis (*Subclade 1b)	74.7–75.8	73.8–74.2	72.4–72.8	73–74.2	90–100			
*T. suis* (Subclade 1a)	72.9–73.8	73.3–74.4	72.1–73.9	73.5–75.1	71.4–72.9	–		
*T. colobae*	70–71.6	72.3	73.3–74.2	73.5–73.9	77.4	78.7	–	
*T. ursinus*	72.1–72.3	72.7–73.3	73.2–74.2	73.5–74.2	75.9–79.1	77.4–77.8	78.5–78-6	99.25–99.8

Subclade TT2a: *Macaca sylvanus* from Spain. Subclade TT2b: *Homo sapiens* from China, *P. anubis* from USA. Subclade TT2c: *M. sylvanus* from Spain, *Macaca fuscata* from Italy, *Papio* sp. from Spain and *P. hamadryas* from Denmark. Subclade TT2d: *M. fuscata* from Italy. Subclade 1a: *Sus scrofa domestica* from Uganda and Denmark and *S.s. scrofa* from Spain. Subclade 1b: *S. s. domestica* from China

## Discussion

Morphological results revealed that the whipworm isolated from *M. sylvanus* is *T. trichiura*. Thus, in agreement with Cutillas et al. [7], Zaman [27] and Tenora et al. [28], the males of this species showed a pair of typical paracloacal papillae. Nevertheless, this is not in agreement with Ooi et al. [6] who reported the existence of a pair of paracloacal papillae associated to a cluster of small papillae not only in *T. trichiura* from human but in males of *T. trichiura* from *M. fuscata* and *Papio papio* and, furthermore, they reported females showing everted vagina covered with sharply pointed spines. We did not observe this type of vagina in *T. trichiura* from *M. sylvanus*. On the other hand, the comparative morphological study carried out on *Trichuris* species from other host primates (*C. guereza* and *P. ursinus*) revealed clear differences in respect to *Trichuris* sp. from *M. sylvanus.* Thus, this can be differentiated from *T. colobae* by the presence of a typical subterminal paracloacal papillae but not associated to a cluster of small papillae and a different spicule to that of *T, colobae* and *T. ursinus*, while females presented a non-everted vagina with a non-ornamented vulva. From a biometrical point of view, a preliminary study [29], based on modern morphometric approach, revealed that the analysis based on three measurements of males (maximum width of the posterior region of the body [thickness, M4], length of the spicule [M8], maximum length of the spicule sheath [M9], clearly illustrates globalized differences in the population of *Trichuris* sp. from *M. sylvanus* showing larger values of the males collected from the macaques with respect to *T. trichiura* from chimpanzees [7, 29]. The occurrence of different biometrical measurements in the same species was explained by Nissen et al. [9] as phenotypic adaptations [1]. This fact was also reported by Cutillas et al. [7, 13]. Furthermore, the existence of different types of eggs of *Trichuris* sp. in the same host has been previously reported [30].

On the other hand, molecular analyses based on mtDNA revealed the existence of two different genotypes corresponding to two different lineages within “*T. trichiura* lineage” that did not correlate with two different morphospecies. Nevertheless, we must be cautious since the number of individuals from one of the populations was very low. This fact would agree with Ghai et al. [31] who found that the host range of *Trichuris* sp. varies depending on the taxonomic group, with some groups showing host specificity and others showing host generality [31]. For this reason, these authors observed that one group was specific to humans, another one had an intermediate host range, and an additional group could infect all primates sampled, including humans. Furthermore, Ravasi et al. [10] found two different genotypes of *Trichuris* sp. from *P. ursinus* from two different geographical locations, but they did not carry out a morphological study to characterize different morphospecies. This morphological study was carried out by Callejón et al. [14] in one of these populations and they described the new species named *T. ursinus* related with *T. suis* lineage.

The combination of certain nuclear and mitochondrial markers could be considered as a useful taxonomic tool in order to infer phylogenetic relationships within *Trichuris* genus. The phylogeny of *Trichuris* spp. from humans and NHP inferred on ribosomal and mitochondrial datasets reported the existence of two main clades previously cited by different authors [10, 14, 32–34]. The similarity between different clades based on DatasetITS2 (“*T. trichiura* lineages” and “*T. suis* lineage”) showed clearly lower value (74–78.6% suggesting that *Trichuris* population of *M. sylvanus* could be considered as *T. trichiura* attending to the intra-population similarity observed) [32].

In addition, phylogenetic relationships within Clade 2 based on ribosomal datasets revealed that phylogenetic relationships of populations of *Trichuris* sp. from *M. sylvanus* were unresolved. Furthermore, *Trichuris* spp. isolated from genus *Macaca* (*M. fuscata*, *M. leonina* and *M. mulatta*) clustered within the same clade separated of *Trichuris* population from *M. sylvanus*. This fact could be explained since *M. sylvanus* is the unique macaque primate extant African representative, all other species being Asiatic suggesting a co-evolutionary process together with the host [35].

The phylogeny inferred on mitochondrial datasets revealed *Trichuris* sp. from *M. sylvanus* (Spain) is separated into two different subclades: TT2a (minority haplotype) and TT2c (majority haplotype). Subclade TT2c is considered the most frequent subclade observed in *Trichuris* spp. from NHP and humans. “*T. trichiura* lineage” included a species complex with hypothetical sibling/cryptic species. In this last lineage, and based on *cox*1 partial gene sequences, *Trichuris* sp. from *M. sylvanus* appeared distributed in two different subclades according to an African or European origin of *T. trichiura* from *H. sapiens*. This phylogenetic pattern of distribution could suggest that different populations are circulating, although samples were taken from the same host. Hawash et al. [36] found no difference between *T. trichiura* from humans and *Trichuris* from NHP in Uganda, and he indicated a specific African parasite origin, which would then has been transmitted to Asia and South America suggesting that *Trichuris* in humans represents an heirloom parasite. We observed that most individuals of *Trichuris* sp. from *M. sylvanus* clustered with *T. trichiura* from *H. sapiens* from Uganda (Africa) and only a few individuals clustered with *Trichuris* sp. of *H. sapiens* from the Czech Republic (Europe). Since only one reference from Africa is used, further molecular studies would be carried out to clarify if there are a specific African parasite origin and a posterior transmission to Europe and Asia.

In agreement with this study, similar results were observed on *Trichuris* sp. from *M. fuscata* [34]. Besides, this population showed two potentially distinct entities of *Trichuris* present in two different subclades: subclade TT2d (analogous to subclade MF reported by Cavallero et al. [34]) and subclade TT2c. These authors suggested the possibility of two different sources of infection for Japanese macaques corresponding with two *Trichuris* taxa. Within Clade 2, subclades TT2a, TT2b and TT2c correspond to taxonomic species able to infect primates and humans without strict host specificity. These results agree with Doležalová et al. [37] revealing the existence of *Trichuris* spp., which are shared by humans and several NHP (baboons and macaques).

Despite the fact that there seems to be a pattern of infection with different *Trichuris* species infecting particular host species, the existence of more species of *Trichuris* in primates opens up the possibility of studying the zoonotic potential of different hosts harboring *T. trichiura* and/or other putative new species of whipworms [31].

In addition, it would be necessary to carry out further morphological and molecular studies of *Trichuris* populations parasitizing NHP from different geographical origins to improve taxonomy and clarify different *Trichuris* species in primates, and to know if the diversity of *Trichuris* spp. parasitizing NHP is due to a host specific process, or if these species share different primate hosts, as well as, to evaluate these primates as reservoir hosts of human trichuriasis.

## Conclusions

The morphological, biometrical, and molecular results showed that adults of *Trichuris* sp. from *M. sylvanus* were *T. trichiura*. Molecular analyses revealed the existence of two different genotypes corresponding to two different lineages within “*T. trichiura* lineage” that did not correspond to different morphospecies.

## Methods

### Isolation of material

Sixty-five adults (32 females and 33 males) of *Trichuris* sp. were collected from the caecum of a male Barbary macaque (*M. sylvanus*), which had died of natural causes, from the Zoo Castellar (Cádiz, Spain). This macaque male was 15-year-old, and it was born in the Zoo Castellar (Cádiz, Spain) and it was in captivity in contact with other individuals of the same species but without contact with others non-human primates’ species. It was in contact with the animal keepers and veterinary from the zoo. The parasitic evaluations revealed the presence of *Trichuris* eggs in the feces for many years and the anthelmintic treatment was mebendazole (10 mg/kg for 3–4 days). A pulmonary pathology was the cause of the natural death.

We previously received consent from the Zoo Castellar to collect these samples. Worms were washed extensively in 0.9% saline solution to remove remains of the host, then, frozen at − 20 °C or preserved in 70% ethanol for morphological, biometrical, and molecular analysis. Posteriorly, worms were cleared with glycerine/alcohol or acetic acid for morphological studies.

### Morphological studies

Species identification was performed according to previous studies [7, 13, 14]. Morphological examinations were carried out as described by Oliveros et al. [38] and Skrjabin et al. [39]. A comparative study of morphological data of *T. trichiura* (present study), *T. colobae* [7, 13] and *T. ursinus* [14] was carried out.

Biometrical analysis of *Trichuris* specimens was carried out according to parameters reported by Spakulová and Lýsek [15], Suriano and Navone [16] and Robles et al. [17]. Subsequently, a biometrical study was carried out using those measurements that are significant in the differentiation of *Trichuris* species and reported previously by García-Sánchez et al. [29]. Descriptive univariate statistics (mean values, standard deviations, and range) for all parameters were determined for all individuals of *Trichuris* sp. from *M. sylvanus*.

We carried out many of the most common tests (including mean, standard deviation, Student’s t) using spreadsheet of Microsoft *Excel*. The Student’s t assess was used to test the equality of means for each variable in both lineages. The following non-redundant measurements (one measurement is not included in another) used for whipworm adults were: Total length, esophagus length (EL), body length (BL), ratio EL/BL, wide body, spicule length, spicule length sheath for male; total length, esophagus length, body length, ratio EL/BL, wide body and vulvar diameter for females [29]. It was considered a value statistically significant when *P* < 0.05. Biometric characters of *Trichuris* sp. from *M. sylvanus* were compared and assayed for a geometric morphometric analysis. Multivariate analyses were used to calculate the phenotypic variations between *Trichuris* specimens, using size-free canonical discriminant analyses on the covariance of log-transformed measurements. These analyses were applied to exclude the effect of within-group ontogenetic variations by reducing the effect of each character on the first-pooled, within-group, principal component (a multivariate size estimator) [39]. The principal component analysis (PCA) was used to summarize most of the variations in a multivariate dataset in a few dimensions. The multivariate analyses of the morphometric data were carried out by using BAC v.2 software [29, 40, 41].

### DNA amplification and sequencing

Genomic DNA from 43 individual was extracted using the DNeasy Blood and Tissue Kit (Qiagen) according to the manufacturer’s protocol. Each nematode was placed in a sterile 1.5 ml Eppendorf tube and a pestle was used to facilitate the mechanic rupture of the cuticle. The genomic DNA was extracted from the complete body. Quality of extractions was assessed using 0.8% agarose gel electrophoresis infused with SYBR® Safe DNA gel stain.

All molecular markers sequenced in the present study (*cox*1 and *co*b, mtDNA and ITS2 rDNA) were amplified by polymerase chain reaction (PCR) using a thermal cycler (Eppendorf AG; Hamburg, Germany). PCR mix, PCR conditions and PCR primers are summarized in the Supporting information (see Additional file 6). The PCR products were checked on SYBR® Safe stained 2% Tris-Borate-EDTA (TBE) agarose gels and purified using the Wizard® SV Gel and PCR Clean-Up System (Promega). The purified PCR products were concentrated and sequenced in both directions using same primers used for PCR by Stab Vida (Portugal).

### Phylogenetic analysis

rDNA (ITS2) and mtDNA (*cox*1 and *co*b) sequences were aligned using the MUSCLE alignment method [26] included in MEGA, version 7.0 [42]. For comparison, additional ribosomal and mitochondrial sequences from *Trichuris* infecting human, NHP and pigs from different geographical regions from the National Centre for Biotechnology Information (NCBI) GenBank™ database were included in the alignments. *Trichinella spiralis* and *Trichinella pseudospiralis* were used as outgroups for mitochondrial datasets (Table 3). No outgroups were used to infer phylogenetic trees based on ITS region because the sequences of nucleotides are not sufficiently conserved for there to be a reasonably unambiguous match. Nevertheless, sequences of *Trichuris* spp. from *Macaca silenus* and *Macaca fascicularis* from the Czech Republic were not included in the phylogenetic analysis due to errors found in these sequences. Moreover, *cox*1 sequences of *Trichuris* spp. from *Macaca mulatta* and *Macaca leonina* were not considered for phylogenetic analysis because they did not correspond with the partial fragment of *cox*1 gene analyzed in the present study; however, they were included in the phylogenetic analysis based on the ITS2 sequence (Table 3).

Phylogenetic analysis was performed by Maximum Parsimony (MP) algorithm using MEGA 7 [42], Maximum Likelihood (ML) using the PHYML package from Guindon and Gascuel [43] and Bayesian Inference (BI) using MrBayes, version 3.2.6 [44]. Each dataset was analyzed separately from each other, and both mitochondrial and ribosomal datasets were combined into a total evidence dataset. jModeltest was employed to compute the best partitioning scheme, as well as the best nucleotide substitution models for each partition [45]. Models of evolution were chosen for subsequent analysis according to the Akaike Information Criterion [46]. The concatenated dataset was partitioned by gene and models for individual genes within partitions were those selected by jModeltest. For ML inference, best-fit nucleotide substitution models included general time reversible (GTR) model with gamma-distributed rate variation and a proportion of invariable sites (GTR + I (*cox*1)), GTR model with gamma-distributed rate variation and a proportion of invariable sites (GTR + I + G (*co*b)) and GTR model with gamma-distributed rate (GTR + G (ITS2)). Support for the topology was examined using bootstrapping (heuristic option) [47] over 1000 replications to assess the relative reliability of clades. The Bayesian posterior probabilities (BPP) comprise the percentage converted for BI; the standard deviation of split frequencies used to determine whether the number of generations completed was enough. Models selected by jModeltest for BI were nst = 6 with inv. rates (*cox*1), nst = 6 with invgamma rates (*co*b) and nst = 6 with gamma rates (ITS2). BI analysis was run for ten million generations, and the tree was sampled every 500 generations. Trees from the first million generations were discarded based on an assessment of convergence. Burn-in was determined empirically by examination of the log likelihood values of the chains. After eliminating the first million trees as “burn-in”, we constructed a 50% majority-rule consensus tree, with nodal values representing the probability (posterior probability) that the recovered clades exist, given the aligned sequence data. We accepted a clade in the Bayesian tree at around 70% posterior probability.

The number of base differences per sequence with respect to the sequences under investigation was evaluated using the number of differences method of MEGA 7 to assess the similarity among all marker sequences of all specimens analyzed in the present study and other *Trichuris* species.

Since molecular analysis showed two different genetic lineages in *Trichuris* sp. from macaque, we carried out a posterior biometrical study based on those measurements and the method previously used considering the two different lineages observed in *Trichuris* sp. from *M. sylvanus*:

- Lineage TT2a: Individuals showing the minority genetic lineage.
- Lineage TT2c: Individuals showing the main genetic lineage.

Thus, the Student’s t test was used to test the equality of means for each variable in both lineages and biometric characters of *Trichuris* sp. from both lineages were compared and assayed for a geometric morphometric analysis.

## Supplementary Information

**Additional file 1. **Biometrical data of 15 males of *Trichuris* sp. isolated from *M. sylvanus.*

**Additional file 2. **Biometrical data of 15 females of *Trichuris* sp. isolated from *M. sylvanus*.

**Additional file 3. **Phylogenetic tree of *Trichuris* species based on *cox*1 mtDNA sequences inferred using Bayesian method. Bayesian Posterior Probabilities of clades are listed first, followed by Maximum Parsimony and Maximum Likelihood bootstrap values, respectively, for clade frequencies exceeding 60%.

**Additional file 4. **Phylogenetic tree of *Trichuris* species based on *co*b mtDNA sequences inferred using Bayesian method. Bayesian Posterior Probabilities of clades are listed first, followed by Maximum Parsimony and Maximum Likelihood bootstrap values, respectively, for clade frequencies exceeding 60%.

**Additional file 5. **Phylogenetic tree of *Trichuris* species based on combined analysis of mitochondrial DNA (*cox*1 and *co*b) and nuclear ribosomal DNA (ITS2) inferred using Bayesian Inference. Bayesian Posterior Probabilities of clades are listed first, followed by Maximum Parsimony and Maximum Likelihood bootstrap values, respectively, for clade frequencies exceeding 65%.

**Additional file 6.** PCR mix, primers and conditions used for each molecular marker sequenced.

## Data Availability

The datasets generated and analyzed during the current study are available in the GenBank™, EMBL and DDBJ repository, [Accession numbers: LR130781–4, LR132031–4, LR535742, LR535746–51] (Table 3).

## Abbreviations

BI: Bayesian inference
bp: base pairs
BPP: Bayesian posterior probabilities
BV: Bootstrap value
*co*b: *cytochrome* b
*cox*1: cytochrome *c-oxidase* subunit 1
DNA: Deoxyribonucleic acid
ITS: Internal transcribed spacer
ML: Maximum likelihood
mm: millimeters
MP: Maximum parsimony
mtDNA: mitochondrial DNA
NHP: Non-human primates
PCA: Principal component analysis
PCR: Polymerase chain reaction
rDNA: ribosomal DNA
