# Correction to: *Trichuris trichiura* isolated from *Macaca sylvanus*: morphological, biometrical, and molecular study

**DOI:** 10.1186/s12917-021-02857-2

**Published:** 2021-04-14

**Authors:** Julia Rivero, Ángela María García-Sánchez, Antonio Zurita, Cristina Cutillas, Rocío Callejón

**Affiliations:** Department of Microbiology and Parasitology, Faculty of Pharmacy, University of Seville, Professor García González 2, 41012 Seville, Spain

**Correction to: BMC Vet Res 16, 445 (2020)**

**https://doi.org/10.1186/s12917-020-02661-4**

*The original article* [1] *contains an incorrect Funding statement which the author would like to change to the following:*

***Funding***

*This work was financially supported by FEDER/Ministry of Science and Innovation - State Research Agency (CGL2017–83057). The Junta de Andalucía (BIO-338), and a grant from the V and VI Plan Propio de Investigación of the University of Seville, Spain*
