# Supplementary material for: Trichuris trichiura isolated from Macaca sylvanus: morphological, biometrical, and molecular study
Source: BMC Vet Res. 2020 Nov 17;16:445. doi: 10.1186/s12917-020-02661-4 (PMC7672873; doi:10.1186/s12917-020-02661-4)
Supplement: Supplementary file 1 — Additional file 1. Biometrical data of 15 males of Trichuris sp. isolated from M. sylvanus. [file 12917_2020_2661_MOESM1_ESM.pdf]

Additional file 1. Biometrical data of 15 males of *Trichuris* sp. isolated from *M. sylvanus*

	**TM1**	**TM2**	**TM3**	**TM4**	**TM5**	**TM6**	**TM7**	**TM8**	**TM9**	**TM10**	**TM11**	**TM12**	**TM13**	**TM14**	**TM15**	**MIN**	**MAX**	**X**	**Б**
**M1**	3.40	3.30	3.80	3.30	3.40	3.90	3.50	3.60	3.80	3.40	3.40	3.40	3.40	3.00	3.10	3.00	3.90	3.45	0.25
**M2**	2.00	2.00	2.80	2.50	2.10	2.50	2.40	2.40	2.10	2.00	2.00	2.00	2.30	1.90	1.90	1.90	2.80	2.19	0.27
**M3**	0.13	0.14	0.13	0.15	0.18	0.12	0.15	0.12	0.15	0.15	0.16	0.15	0.15	0.16	0.12	0.12	0.18	0.14	0.02
**M4**	0.74	0.58	0.62	0.52	0.64	0.63	0.64	0.63	0.56	0.53	0.61	0.64	0.69	0.67	0.49	0.49	0.74	0.61	0.07
**M5**	0.49	0.37	0.29	0.35	0.41	0.40	0.38	0.33	0.40	0.33	0.39	0.42	0.43	0.32	0.25	0.25	0.49	0.37	0.06
**M6**	0.44	0.43	0.64	0.45	0.52	0.59	0.53	0.46	-	0.50	0.60	0.40	0.33	0.40	0.63	0.33	0.64	0.49	0.09
**M7**	1.26	1.18	1.23	1.37	1.47	1.57	1.49	1.29	-	1.17	1.32	1.45	1.27	1.38	1.10	1.10	1.57	1.33	0.14
**M8**	2.52	2.23	2.72	2.58	2.72	2.75	2.70	2.61	2.58	2.43	2.55	2.55	3.23	2.91	2.63	2.23	3.23	2.65	0.23
**M9**	1.03	0.76	1.23	1.05	1.14	0.84	1.15	1.05	0.90	1.08	0.53	0.70	0.87	0.94	0.71	0.53	1.23	0.93	0.20
**M10**	0.08	0.08	0.07	0.06	0.06	0.05	0.04	0.07	0.05	0.06	0.07	-	0.05	0.07	0.08	0.04	0.08	0.06	0.01
**M11**	0.08	0.06	0.07	0.04	0.04	0.05	0.07	0.04	0.05	0.04	0.05	0.08	0.05	-	0.05	0.04	0.08	0.06	0.01
**M12**	0.08	0.06	0.07	0.06	0.06	0.06	0.07	0.07	0.06	0.06	0.07	-	0.06	0.09	0.06	0.06	0.09	0.07	0.01
**M13**	2.90	3.19	3.60	4.42	4.22	4.66	5.19	4.60	4.03	3.78	3.88	3.99	4.19	3.84	4.34	2.90	5.19	4.06	0.58
**M14**	1.98	1.76	1.47	1.96	1.99	2.29	2.34	2.23	1.93	2.23	1.32	2.24	2.07	1.84	1.85	1.32	2.34	1.97	0.29
**M15**	2.31	1.65	2.34	2.52	2.75	2.34	2.37	2.37	2.09	1.55	2.44	1.55	2.12	2.00	2.49	1.55	2.75	2.20	0.36

All measurements are in millimetres. M1: total body length of adult worm. M2: length of oesophageal region of body. M3: width of oesophageal region of body. M4 maximum width of posterior region of body (thickness). M5: body width in the place of junction of oesophagus and the intestine. M6: distance from the head end to the beginning of bacillary stripes. M7: length of bacillary stripes. M8: length of spicule. M9: maximum length of spicule sheath. M10: width of proximal end of spicule. M11: width of spicule sheath at the tail end of body. M12: maximum width of spicule sheath. M13: length of anterior region of cloaca. M14: length of ejaculatory duct. M15: distance between posterior part of testis and tail end of body. Б: Standard deviation. X: Arithmetic mean. Min: Minimum value; Max: Maximum value.
