# Supplementary material for: Trichuris trichiura isolated from Macaca sylvanus: morphological, biometrical, and molecular study
Source: BMC Vet Res. 2020 Nov 17;16:445. doi: 10.1186/s12917-020-02661-4 (PMC7672873; doi:10.1186/s12917-020-02661-4)
Supplement: Supplementary file 2 — Additional file 2. Biometrical data of 15 females of Trichuris sp. isolated from M. sylvanus. [file 12917_2020_2661_MOESM2_ESM.pdf]

Additional file 2. Biometrical data of 15 females of *Trichuris* sp. isolated from *M. sylvanus*

	**TF1**	**TF2**	**TF3**	**TF4**	**TF5**	**TF6**	**TF7**	**TF8**	**TF9**	**TF10**	**TF11**	**TF12**	**TF13**	**TF14**	**TF15**	**MIN**	**MAX**	**X**	**Б**
**F1**	3.30	3.60	3.20	3.10	3.10	3.50	3.60	3.20	3.60	3.80	3.60	3.70	3.50	3.00	3.30	3.00	3.80	3.41	0.25
**F2**	2.20	2.30	2.30	2.00	1.80	2.10	2.20	1.90	2.30	2.60	2.20	2.60	2.20	1.90	2.30	1.80	2.60	2.19	0.23
**F3**	0.13	0.15	0.16	0.15	0.16	0.15	0.16	0.15	0.18	0.15	0.15	0.14	0.15	0.16	0.16	0.13	0.18	0.15	0.01
**F4**	0.70	0.67	0.70	0.77	0.70	0.72	0.73	0.69	0.78	0.81	0.73	0.78	0.65	0.66	0.64	0.64	0.81	0.72	0.05
**F5**	0.42	0.39	0.36	0.42	0.42	0.46	0.42	0.44	0.48	0.42	0.42	0.44	0.40	0.41	0.39	0.36	0.48	0.42	0.03
**F6**	0.52	0.42	0.45	0.42	0.56	0.46	0.47	0.48	0.49	0.53	0.46	0.49	0.76	0.56	0.43	0.42	0.76	0.50	0.09
**F7**	1.67	1.52	1.52	1.64	0.90	1.15	1.52	1.31	1.41	1.71	1.35	1.63	1.47	1.33	1.53	0.90	1.71	1.44	0.21
**F8**	1.82	1.11	1.11	0.76	0.91	0.81	1.05	0.73	0.88	1.11	1.17	1.00	1.16	1.11	1.99	0.73	1.99	1.12	0.35
**F9**	0.06	0.05	0.04	0.02	0.06	0.05	0.03	0.04	0.06	0.09	0.04	0.05	0.04	0.06	0.06	0.02	0.09	0.05	0.02
**F10**	0.24	0.23	0.23	0.21	0.22	0.22	0.33	0.15	0.29	0.26	0.26	0.23	0.27	0.32	0.23	0.15	0.33	0.25	0.05
**F11**	0.67	0.61	0.67	0.62	-	0.67	0.68	0.52	0.71	0.51	0.83	0.84	0.46	0.42	0.40	0.40	0.84	0.61	0.14
**F12**	0.21	0.41	0.48	0.24	0.19	0.26	0.22	0.36	0.29	0.29	0.21	0.40	0.38	0.20	0.40	0.19	0.48	0.30	0.09
**F13**	0.14	0.14	0.10	-	0.13	0.11	-	-	-	-	-	0.05	-	-	-	0.05	0.14	0.11	0.04

All measurements are in millimetres. F1: Total body length of adult worm; F2: Length of oesophageal region of body; F3: Width of esophageal region of body; F4: Maximum width of posterior region of body (thickness); F5: Body width in the place of junction of oesophagus and the intestine; F6: Distance from the head end to beginning of bacillary stripes; F7: Length of bacillary stripes; F8: Length of vagina; F9: Diameter of vulva turned over the surface of body; F10: Distance of vulva from place of junction of oesophagus and the intestine; F11: Distance of posterior loop of uterus from tail end of body; F12: Distance of tail end of body and posterior fold of seminal receptacle; F13: Length of muscular zone of the oesophagus. Б: standard deviation. X: arithmetic mean. Min: Minimum value; Max: Maximum value.
