# Supplementary figures and images for: Trichuris trichiura isolated from Macaca sylvanus: morphological, biometrical, and molecular study
Source: BMC Vet Res. 2020 Nov 17;16:445. doi: 10.1186/s12917-020-02661-4 (PMC7672873; doi:10.1186/s12917-020-02661-4)

Additional file 3

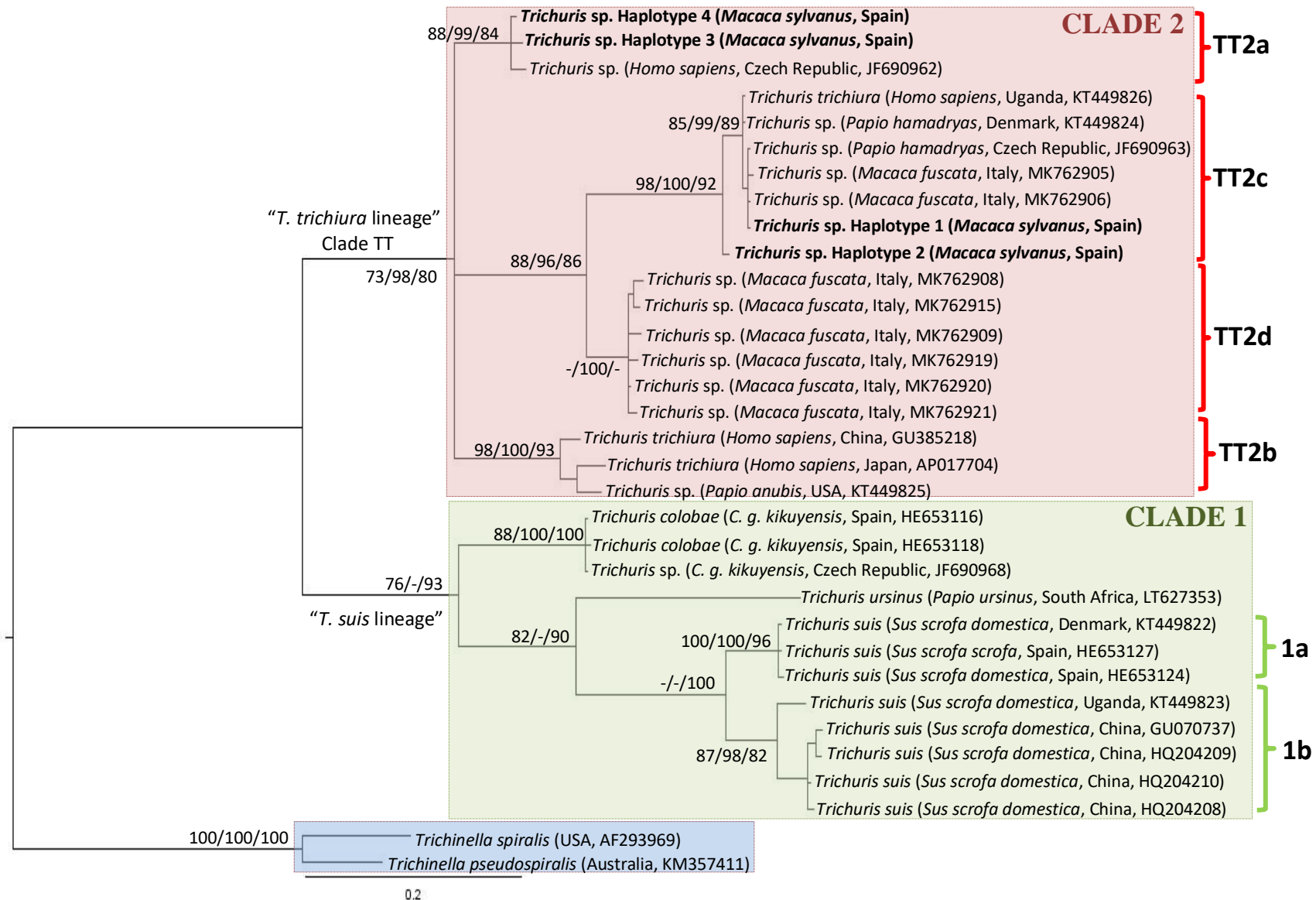

Supplement: Supplementary file 3 — Additional file 3. Phylogenetic tree of Trichuris species based on cox1 mtDNA sequences inferred using Bayesian method. Bayesian Posterior Probabilities of clades are listed first, followed by Maximum Parsimony and Maximum Likelihood bootstrap values, respectively, for clade frequencies exceeding 60%. [file 12917_2020_2661_MOESM3_ESM.pdf]

## Additional file 4

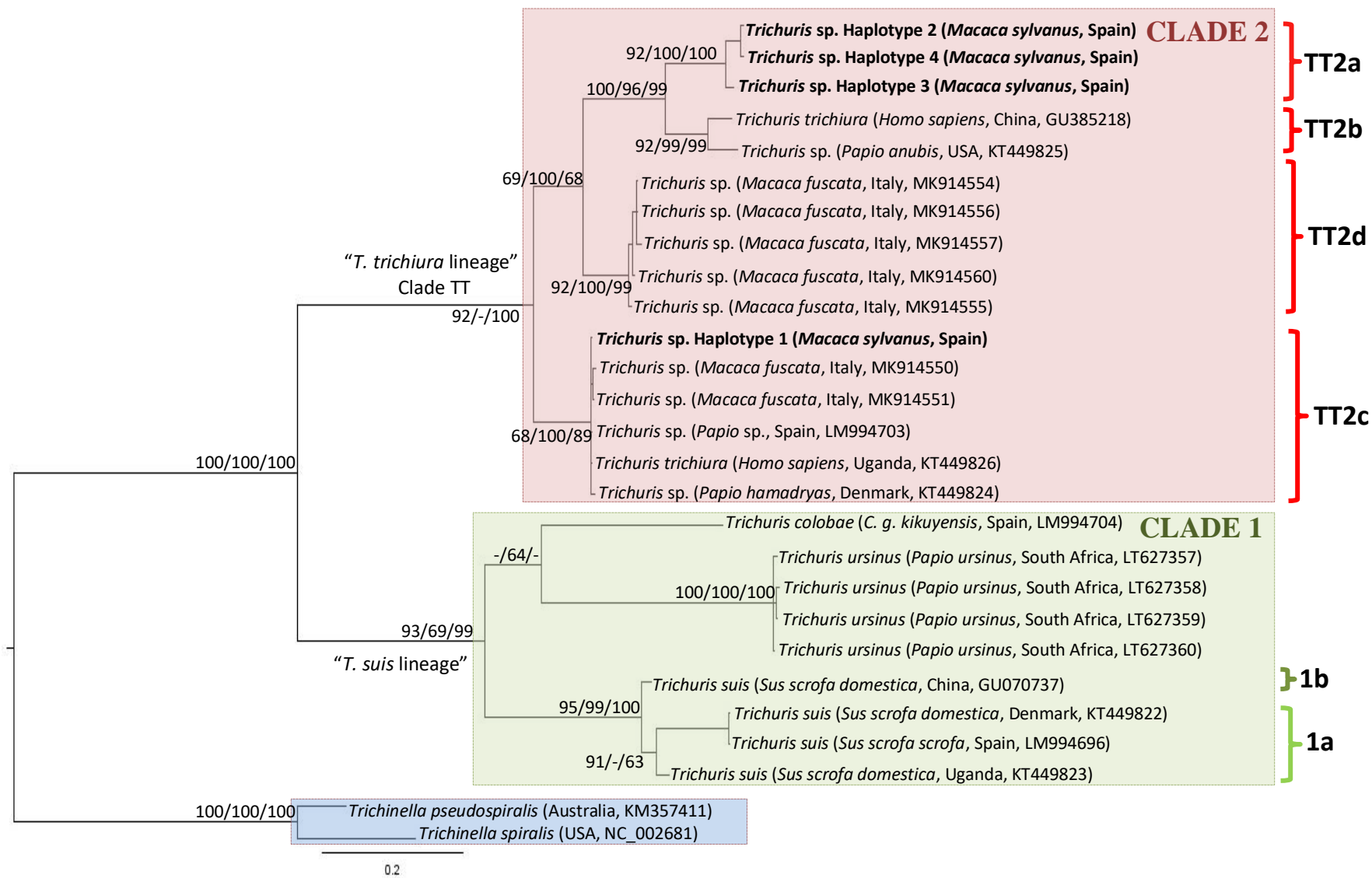

Supplement: Supplementary file 4 — Additional file 4. Phylogenetic tree of Trichuris species based on cob mtDNA sequences inferred using Bayesian method. Bayesian Posterior Probabilities of clades are listed first, followed by Maximum Parsimony and Maximum Likelihood bootstrap values, respectively, for clade frequencies exceeding 60%. [file 12917_2020_2661_MOESM4_ESM.pdf]

Additional file 5

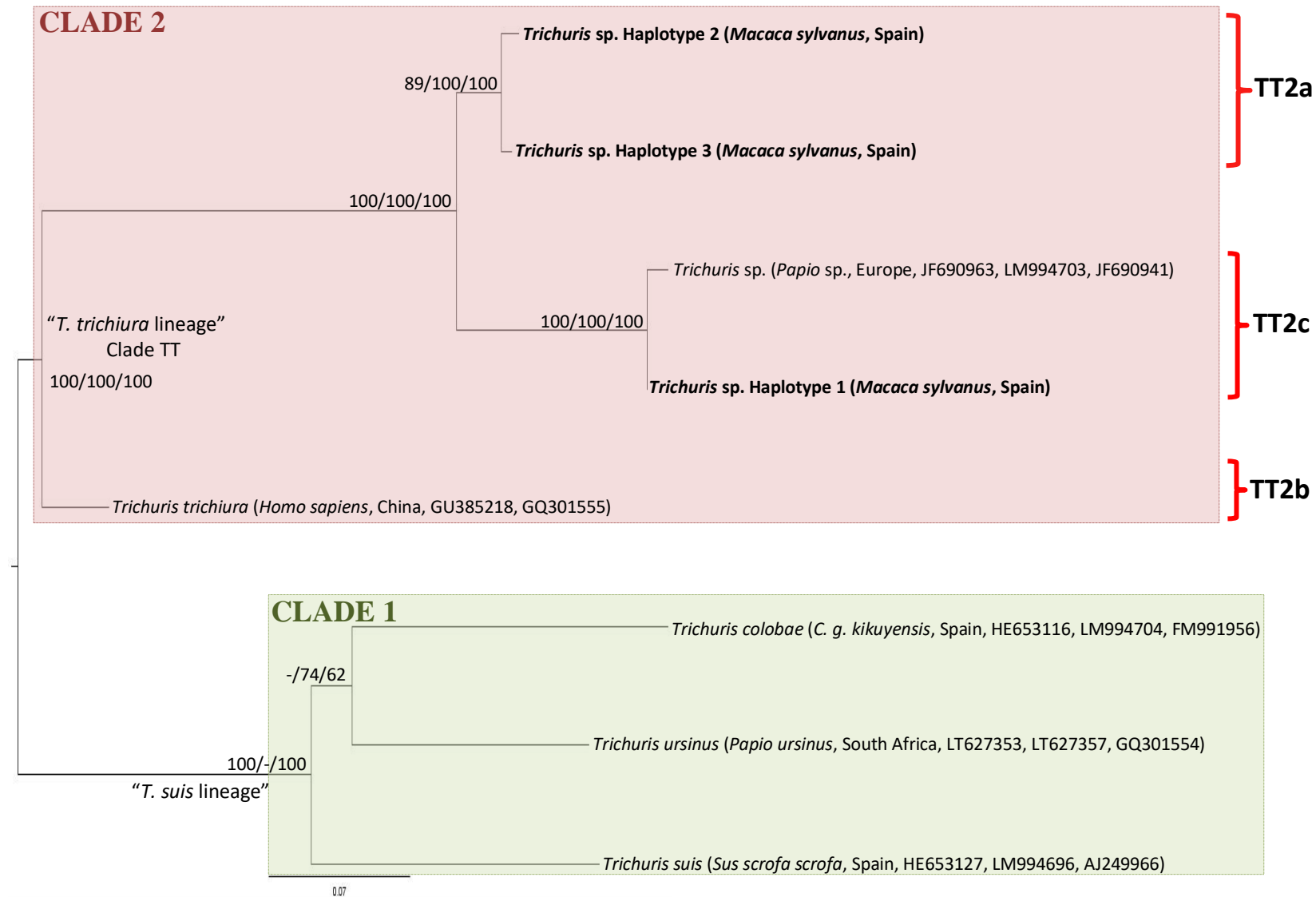

Supplement: Supplementary file 5 — Additional file 5. Phylogenetic tree of Trichuris species based on combined analysis of mitochondrial DNA (cox1 and cob) and nuclear ribosomal DNA (ITS2) inferred using Bayesian Inference. Bayesian Posterior Probabilities of clades are listed first, followed by Maximum Parsimony and Maximum Likelihood bootstrap values, respectively, for clade frequencies exceeding 65%. [file 12917_2020_2661_MOESM5_ESM.pdf]
