# Supplementary material for: Trichuris trichiura isolated from Macaca sylvanus: morphological, biometrical, and molecular study
Source: BMC Vet Res. 2020 Nov 17;16:445. doi: 10.1186/s12917-020-02661-4 (PMC7672873; doi:10.1186/s12917-020-02661-4)
Supplement: Supplementary file 6 — Additional file 6. PCR mix, primers and conditions used for each molecular marker sequenced. [file 12917_2020_2661_MOESM6_ESM.pdf]

Additional file 6. PCR mix, primers and conditions used for each molecular marker sequenced in the present study.

	ITS2	*Cox1*	*Cob*
	**PCR Mix**		
**Forward Primer (10 µM)**	5 µl	5 µl	5 µl
**Reverse Primer (10 µM)**	5 µl	5 µl	5 µl
**GoTaq G2 Green Master Mix**	25 µl	25 µl	25 µl
**Template DNA**	5 µl	5 µl	5 µl
**Nuclease free water to**	50 µl	50 µl	50 µl
	**PCR Primers**		
**Forward Primer**	5.8SF (Robles et al., 2014)	HC02198F (Folmer et al., 1994)	D769 (Callejón et al., 2015)
**Reverse Primer**	ITS2R (Ellis and Horvitz, 1986)	CORA (Nagano et al., 1999)	D770 (Callejón et al., 2015)
	**PCR Conditions**		
**Initial Denaturing**	94 °C/3 min	94 °C/5 min	94 °C/5 min
**Number of cycles**	35	40	36
**Denaturing**	94 °C/1 min	94 °C/1min	94 °C/30 s
**Annealing**	50 °C/1 min	48 °C/1min	50 °C/30 s
**Primer extension**	72 °C/1 min	72 °C/1min	72 °C/30 s
**Final extension**	72 °C/10 min	72 °C/7min	72 °C/5 min
